# Severe hypoglycemia and the risk of cardiovascular disease and mortality in type 2 diabetes: a nationwide population-based cohort study

**DOI:** 10.1186/s12933-019-0909-y

**Published:** 2019-08-14

**Authors:** Jae-Seung Yun, Yong-Moon Park, Kyungdo Han, Seon-Ah Cha, Yu-Bae Ahn, Seung-Hyun Ko

**Affiliations:** 1 Division of Endocrinology and Metabolism, Department of Internal Medicine, St. Vincent’s Hospital, College of Medicine, The Catholic University of Korea, Seoul, South Korea; 2 Epidemiology Branch, National Institute of Environmental Health Sciences, National Institutes of Health, Research Triangle Park, NC USA; 3 Department of Biostatistics, College of Medicine, The Catholic University of Korea, Seoul, South Korea

**Keywords:** Cardiovascular disease, Severe hypoglycemia, Type 2 diabetes

## Abstract

**Background:**

We investigated the association regarding severe hypoglycemia episodes with cardiovascular disease risk and all-cause mortality in patients with type 2 diabetes.

**Methods:**

Baseline and follow-up data (n = 1,568,097) from patients with type 2 diabetes were retrieved from the National Health Insurance System database (covering the entire Korean population). Type 2 diabetes, severe hypoglycemia, and major comorbidities were identified using International Classification of Diseases 10 codes and medication information. Individuals who were classified as type 2 diabetes in the year of 2009 were screened, and we counted severe hypoglycemia episodes from 2007 to 2009. The primary outcome was newly developed myocardial infarction (MI), stroke, heart failure, or all-cause mortality. Participants were followed from the baseline index date to the date of death or until December 31, 2015.

**Results:**

In total, 19,660 (1.2%) patients developed at least one severe hypoglycemia event during the period from 2007 to 2009. Mean follow-up was 5.7 years. After adjustment for confounding factors, the hazard ratio (HR) of MI significantly and sequentially increased: 0 vs. 1 episode, HR 1.56, 95% CI 1.46–1.64; 0 vs. 2 episodes, HR 1.86, 95% CI 1.61–2.15; 0 vs. 3 or more episodes, HR 1.86, 95% CI 1.48–2.35, *P* for trend < 0.001. Similar findings were noted regarding the relationship of severe hypoglycemia episodes with stroke, heart failure, and all-cause mortality. Risks for all outcomes were highest within 1 year from the index date and showed decreasing trends with follow-up. Sensitivity analyses of the data from the subgroup population and 797,544 subjects who received a national health examination did not change the significance of the main findings.

**Conclusion:**

Among adult Korean patients with type 2 diabetes, a severe hypoglycemia episode is associated with increased risk for cardiovascular outcomes and all-cause mortality. Significant results from dose–response, temporal, and sensitivity analyses may suggest the possibility of direct causality between severe hypoglycemia and cardiovascular outcomes and mortality.

**Electronic supplementary material:**

The online version of this article (10.1186/s12933-019-0909-y) contains supplementary material, which is available to authorized users.

## Background

A recent report from the International Diabetes Federation suggested the global prevalence of diabetes mellitus in adults is 1 in 11; this indicates that diabetes has become a silent epidemic on a global scale and poses a serious health concern [1]. Poor glycemic control has contributed tremendously to the burden of diabetic complications and mortality, especially those related with cardiovascular disease (CVD). However, studies concerning treatment intensification have failed to demonstrate an improved risk for CVD, and there are concerns about the risks of aggressive treatment, including hypoglycemia [2, 3]. Thus, many clinical trials suggested that more aggressive glycated hemoglobin (HbA1c) and glycemic target goals should be developed based on the anticipated benefits and risk of severe hypoglycemia (SH) [4].

SH, which is defined as a hypoglycemic episode requiring aid to treat, is associated with a wide range of adverse outcomes [5]. Several analyses suggest that hypoglycemia may be associated with CVD and mortality in type 2 diabetes [6–12]. When a hypoglycemic episode occurs, there may be an increase in sympathetic activity which can cause destabilization of atherosclerotic plaques and lead to cardiac arrhythmia, vascular dysfunction, and inflammation [13, 14]. However, it is also possible that individuals prone to hypoglycemic episodes may have another attribute that places them at risk for poor cardiovascular outcomes. Thus, there is an unresolved, long-standing debate regarding whether SH events are merely markers of vulnerability or whether they play a causal role in the development of CVD events and mortality [7, 8, 15–17]. In addition, there is a lack of consistent evidence concerning the dose–response and temporal relationship between the frequency of SH episodes and cardiovascular outcomes in type 2 diabetes [11, 12, 18].

To address this, our study aim was to evaluate the association between SH and myocardial infarction, stroke, heart failure, and all-cause mortality in type 2 diabetes using the National Health Insurance Service (NHIS) database, which covers the entire Korean population through a social insurance benefit system [19]. We also aimed to clarify the association between the frequency and timing of the SH events and the cardiovascular outcomes.

## Methods

### Data source

**Fig. 1:**
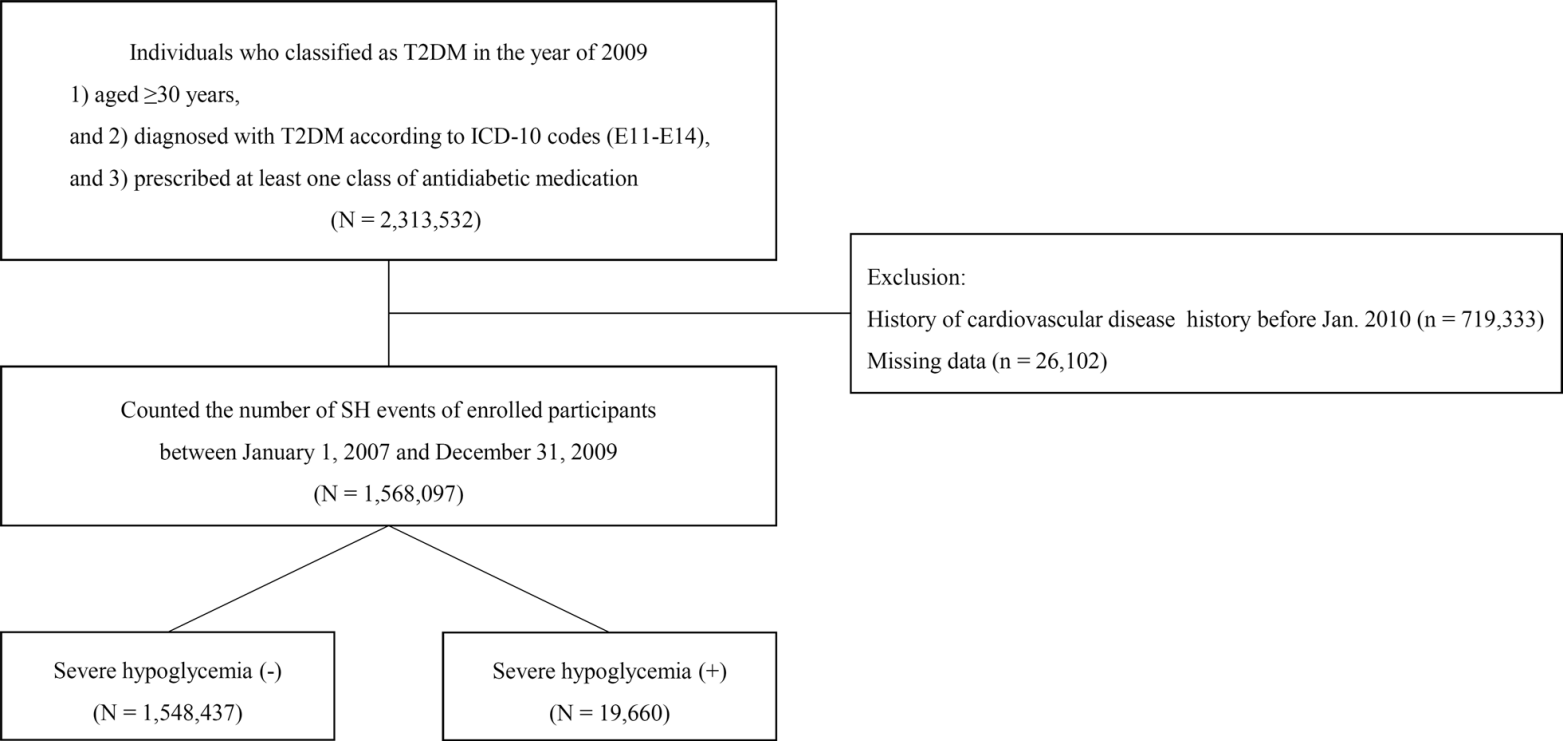
Sample recruitment from the database of National Health Insurance Service

We performed a nationwide retrospective study using the Korean National Health Insurance (NHI) Claims Database maintained by the NHIS, a government-affiliated agency that administers the medical service system in Korea. This database includes general information on specifications, drug prescription information, consultation statement, and diagnosis statement using International Classification of Disease, 10th revision (ICD-10) classifications [20]. In addition, all adult Korean people aged 30 years and older are encouraged to perform regular standardized general medical checkups including a questionnaire for smoking status, alcohol consumption, measurements of height, weight, and laboratory tests once every 2 years [20]. To protect individual privacy, each subjects’ information was anonymized. This study was approved by the appropriate institutional review board (approval number: VC17RESI0160) and conducted in compliance with the Declaration of Helsinki. Participants were followed from the baseline index date to the date of death or until December 31, 2015 (Fig. 1).

### Study population

Among all NHI beneficiaries, participants aged 30 years and older and who were diagnosed with type 2 diabetes according to ICD-10 codes (E11–E14) in the year of 2009 were included at baseline. Considering the characteristics of the Korean NHIS database, an operational definition of diabetes described in detail elsewhere was applied in this study [19]. These patients made at least one visit to outpatient or inpatient care for type 2 diabetes diagnosis and were prescribed at least one class of antidiabetic medication. Exclusion criteria were type 1 diabetes, gestational diabetes, missing data, and diagnosis with cardiovascular disease, including MI, stroke, and congestive heart failure before the index date.

### Definition of variables

Due to the limitation of claims data, we collected all episodes reporting SH, which was defined as the diagnostic clinical codes for hypoglycemia (ICD-10 codes of E 16.x, E1163, E1363, and E1463) from the inpatient or emergency room claims dataset, and we counted the number of SH events of enrolled participants between January 1, 2007 and December 31, 2009. The index date was defined as January 1, 2010. The primary endpoint of this study was newly first diagnosed myocardial infarction (I21-I22), stroke (I63-I64), and heart failure (I50), based on codes from the ICD-10, along with subsequent hospitalization during the follow-up period. Claims data and national mortality data from the National Statistical Office were merged for analysis of death.

The criteria for the socioeconomic status were based on the average monthly insurance payments imposed by the Korean National Health Insurance Corporation, which are determined based on annual income level in Korea. Socioeconomic status was categorized into three groups (lower 30%; mid 40%; upper 30%) based on income levels. Anti-diabetic drugs included seven classes (biguanide, sulfonylurea, a-glucosidase inhibitor, thiazolidinedione, meglitinide, dipeptidyl peptidase-4 inhibitor, and insulin) dispensed at the baseline period in Korea. Hypertension was defined using the ICD-10 code (I10-I13, I15) and prescription of anti-hypertensive drugs. The subjects’ medical history, including chronic obstructive pulmonary disease (COPD, J43–J44), all types of cancer (C00–C97), liver cirrhosis (K704, K746), and end-stage renal disease (ESRD, N18, N19, Z49, Z905, Z94, and Z992 of ICD-10 code and R380, O7011–7020, O7017, and O7075 of the procedure code), was identified using the ICD-10 codes. These covariates and baseline characteristics were defined using the data from the year 2009. In the sub-cohort for sensitivity analysis that consisted of the participants who received regular medical check-ups, information concerning smoking status (never smoked; former smoker; current smoker), drinking habit (never drank alcohol; moderate drinker, ≤ 1 drink per day; heavy drinker, > 1 drink per day), and physical activity (no exercise, 1–2 times per week, ≥ 3 times per week) was obtained using a standard questionnaire during the health examination. Body mass index (BMI) was calculated as weight in kilograms divided by the square of height in meters (kg/m^2^). Estimated glomerular filtration rate (eGFR) was calculated from serum creatinine using the Modification of Diet in Renal Disease Study Group equation (MDRD). Chronic kidney disease was defined as an eGFR < 60 mL/min/1.73 m^2^. Hypertension was defined by claim codes (ICD-10, I10-I13 and I15 and prescription of anti-hypertensive agents or systolic or diastolic blood pressure ≥ 140 and ≥ 90 mmHg). Dyslipidemia was defined by claim codes (ICD-10, E78 and prescription of lipid lowering agents or total cholesterol ≥ 6.2 mmol/L). The covariates and baseline characteristics of regular medical checkup data were defined or measured from the results of the National Health Examination Program in the 2008–2009 year.

### Statistical analysis

The demographic characteristics for the participants were analyzed using descriptive statistics. Data are expressed as the mean ± standard deviation for normally distributed continuous variables and as proportions for categorical variables. The characteristics of the groups according to the number of SH events were compared using a one-way analysis of variance or the Chi squared test. Firstly, we analyzed subjects from the entire population in Korea, which included the subjects who partly lacked general medical checkup data. Secondly, we analyzed the sub-cohort from the entire population who had received a health examination at least once between the year of 2008 and 2009 for validation. We evaluated the association of the number of severe hypoglycemia (0, 1, 2, 3 or more) episodes with cardiovascular outcomes and all-cause mortality using Cox proportional hazard regression analysis. We also adjusted the models for potential confounders. Model 1 was a crude analysis, Model 2 was a sex- and age-adjusted analysis, and Model 3 was a further multivariable analysis adjusted for the following variables: age, sex, living place (urban or rural), income level, anti-diabetic drugs, the presence of hypertension, dyslipidemia, and major comorbidities (malignancy, liver cirrhosis, ESRD, and COPD). We performed a comparison of baseline and hazard ratio values using a trend analysis. The trends of values across the increasing outcomes were estimated using a multiple linear regression analysis for continuous variables and a Chi square test for categorical variables. Age was adjusted as a continuous variable. Given that mortality could compete with cardiovascular outcomes of interest, we performed a competing risk analysis using a sub-distribution hazards model [21].

Hazard ratios (HR) and 95% confidence intervals (CI) for the Cox proportional hazard model were used to evaluate the association between SH episodes and main outcomes. To confirm the time effect (temporality) of SH events on the risk of main outcomes, we compared the results by dividing the follow-up period into three categories 12 months or less, 13–24 months, and over 24 months from the index date. A sensitivity analysis was performed for the sub-cohort, which included only those who received the standardized medical checkup. Variables that could only be obtained from the medical health check-up database, such as smoking status, alcohol consumption, income level by standardized self-reporting questionnaires, BMI, blood pressure, and laboratory test results, were included in the sensitivity analysis. Forest plots for incident main outcomes in subgroups were evaluated. All statistical analyses were performed using Statistical Package for SAS 9.2. *P* value, *P* for trend, and *P* for interaction < 0.05 were considered statistically significant.

## Results

**Fig. 2:**
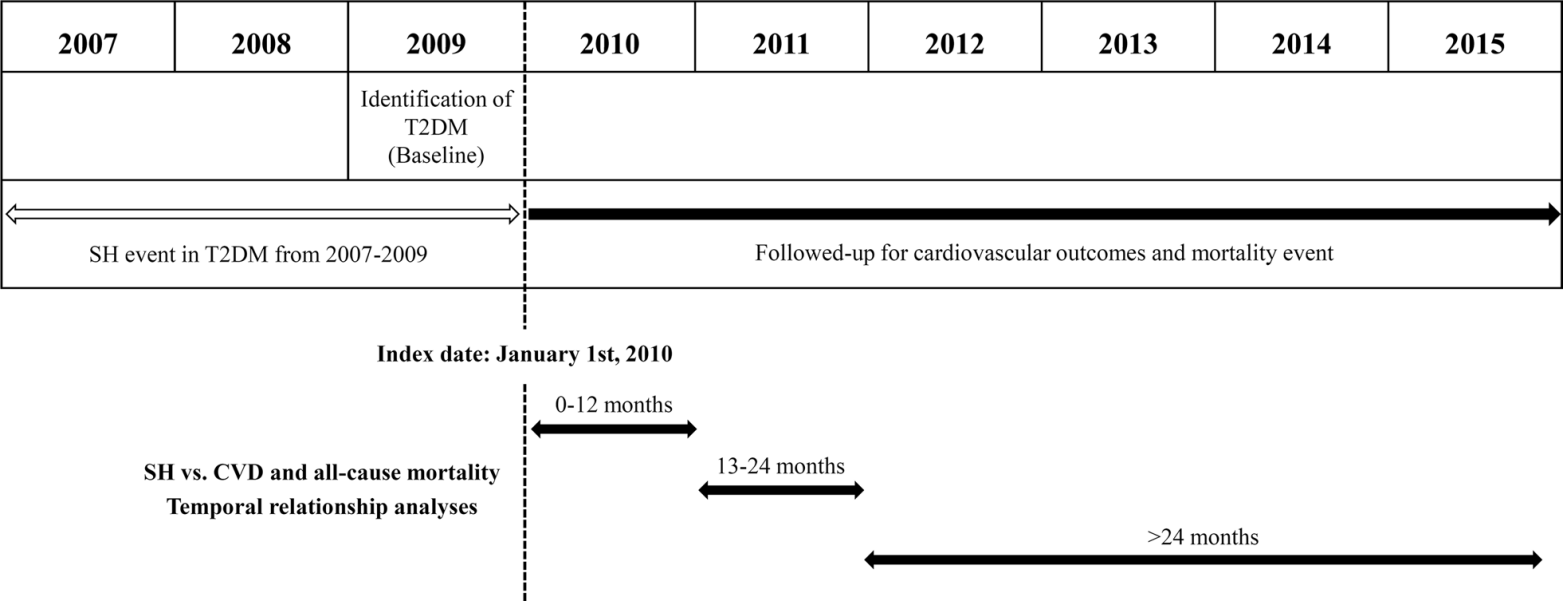
Study design summarizing subject selection and follow-up

Of the 2,287,430 participants who had type 2 diabetes in the year of 2009, a total of 1,568,097 participants were followed from baseline to the end of the study period, with a mean follow-up duration of 5.7 years (Fig. 2). The mean age of subjects was 58.6 ± 11.5 years, 54.7% of whom were men. Among participants with diagnosed type 2 diabetes, 19,660 (1.2%) experienced SH, and 3668 (0.3%) experienced at least two or more episodes of SH during the baseline period (2007–2009). Participants who experienced SH at baseline were more likely to be older, female, and to use insulin. They were also more likely to be in the lower income group compared with participants who did not experience SH. Participants who experienced SH had a higher ratio of hypertension, more major comorbidities, and a lower rate of dyslipidemia (Table 1).

**Table 1:** Baseline characteristics according to the number of severe hypoglycemia event

	Total	Severe hypoglycemia			Number of severe hypoglycemia				
		No	Yes	*P* value	No	1	2	3 or more	*P* for trend
Total	1,568,097	1,548,437 (98.7)	19,660 (1.3)		1,548,437 (98.7)	15,992 (1.0)	2543 (0.2)	1125 (0.1)	
Age (%)				< 0.001					< 0.001
30–49 years	360,764 (23.0)	358,253 (23.1)	2511 (12.8)		358,253 (23.1)	1985 (12.4)	309 (12.2)	217 (19.3)	
50–64 years	681,133 (43.4)	675,499 (43.6)	5634 (28.7)		675,499 (43.6)	4715 (29.5)	627 (24.7)	292 (26.0)	
65– years	526,200 (33.6)	514,685 (33.2)	11,515 (58.6)		514,685 (33.2)	9292 (58.1)	1607 (63.2)	616 (54.8)	
Sex (male)	857,078 (54.7)	847,922 (54.8)	9156 (46.6)	< 0.001	847,922 (54.8)	7394 (46.2)	1181 (46.4)	581 (51.6)	< 0.001
Urban (%)	737,077 (47.0)	729,017 (47.1)	8060 (41.0)	< 0.001	729,017 (47.1)	6664 (41.7)	980 (38.6)	416 (37.0)	< 0.001
Socioeconomic status (%)				< 0.001					< 0.001
Lower 30%	533,293 (34.0)	524,963 (33.9)	8330 (42.4)		524,963 (33.9)	6530 (40.8)	1179 (46.4)	621 (55.2)	
Mid 40%	486,963 (31.1)	481,579 (31.1)	5384 (27.4)		481,579 (31.1)	4508 (28.2)	640 (25.2)	236 (21.0)	
Upper 30%	547,841 (34.9)	541,895 (35.0)	5946 (30.2)		541,895 (35.0)	4954 (31.0)	724 (28.5)	268 (23.8)	
Medication (%)									
Insulin	224,761 (14.3)	215,976 (14.0)	8785 (44.7)	< 0.001	215,976 (14.0)	6800 (42.5)	1316 (51.8)	669 (59.5)	< 0.001
Sulfonylurea	1223,327 (78.0)	1,208,722 (78.1)	14,605 (74.3)	< 0.001	1,208,722 (78.1)	12,043 (75.3)	1827 (71.8)	735 (65.3)	< 0.001
Metformin	1,110,628 (70.8)	1,097,712 (70.9)	12,916 (65.7)	< 0.001	1,097,712 (70.9)	10,562 (66.1)	1664 (65.4)	690 (61.3)	< 0.001
Meglitinide	74,406 (4.7)	72,350 (4.67)	2056 (10.5)	< 0.001	72,350 (4.67)	1571 (9.8)	346 (13.6)	139 (12.4)	< 0.001
Thiazolidinedione	212,955 (13.6)	210,178 (13.6)	2777 (14.1)	0.025	210,178 (13.6)	2274 (14.2)	344 (13.5)	159 (14.1)	0.115
DPP4 inhibitor	137,010 (8.7)	135,428 (8.8)	1582 (8.1)	0.001	135,428 (8.8)	1265 (7.9)	224 (8.8)	93 (8.3)	0.003
Acarbose	345,942 (22.1)	339,257 (21.9)	6685 (34.0)	< 0.001	339,257 (21.9)	5330 (33.3)	968 (38.1)	387 (34.4)	< 0.001
Hypertension (%)	872,321 (55.6)	858,893 (55.5)	13,428 (68.3)	< 0.001	858,893 (55.5)	10,895 (68.1)	1778 (69.9)	755 (67.1)	< 0.001
Dyslipidemia (%)	593,295 (37.8)	586,718 (37.9)	6577 (33.5)	< 0.001	586,718 (37.9)	5496 (34.4)	791 (31.1)	290 (25.8)	< 0.001
COPD (%)	52,126 (3.3)	51,244 (3.3)	882 (4.5)	< 0.001	51,244 (3.3)	712 (4.5)	124 (4.9)	46 (4.1)	< 0.001
Malignancy (%)	17,121 (1.1)	16,738 (1.1)	383 (2.0)	< 0.001	16,738 (1.1)	312 (2.0)	52 (2.0)	19 (1.7)	< 0.001
Liver cirrhosis (%)	4072 (0.3)	3984 (0.3)	88 (0.5)	< 0.001	3984 (0.3)	71 (0.4)	12 (0.5)	5 (0.4)	< 0.001
ESRD (%)	962 (0.1)	862 (0.1)	100 (0.5)	< 0.001	862 (0.1)	79 (0.5)	13 (0.5)	8 (0.7)	< 0.001

Values are presented as percentage or mean ± standard deviation

*COPD* chronic obstructive pulmonary disease, *ESRD* end-stage renal disease

**Fig. 3:**
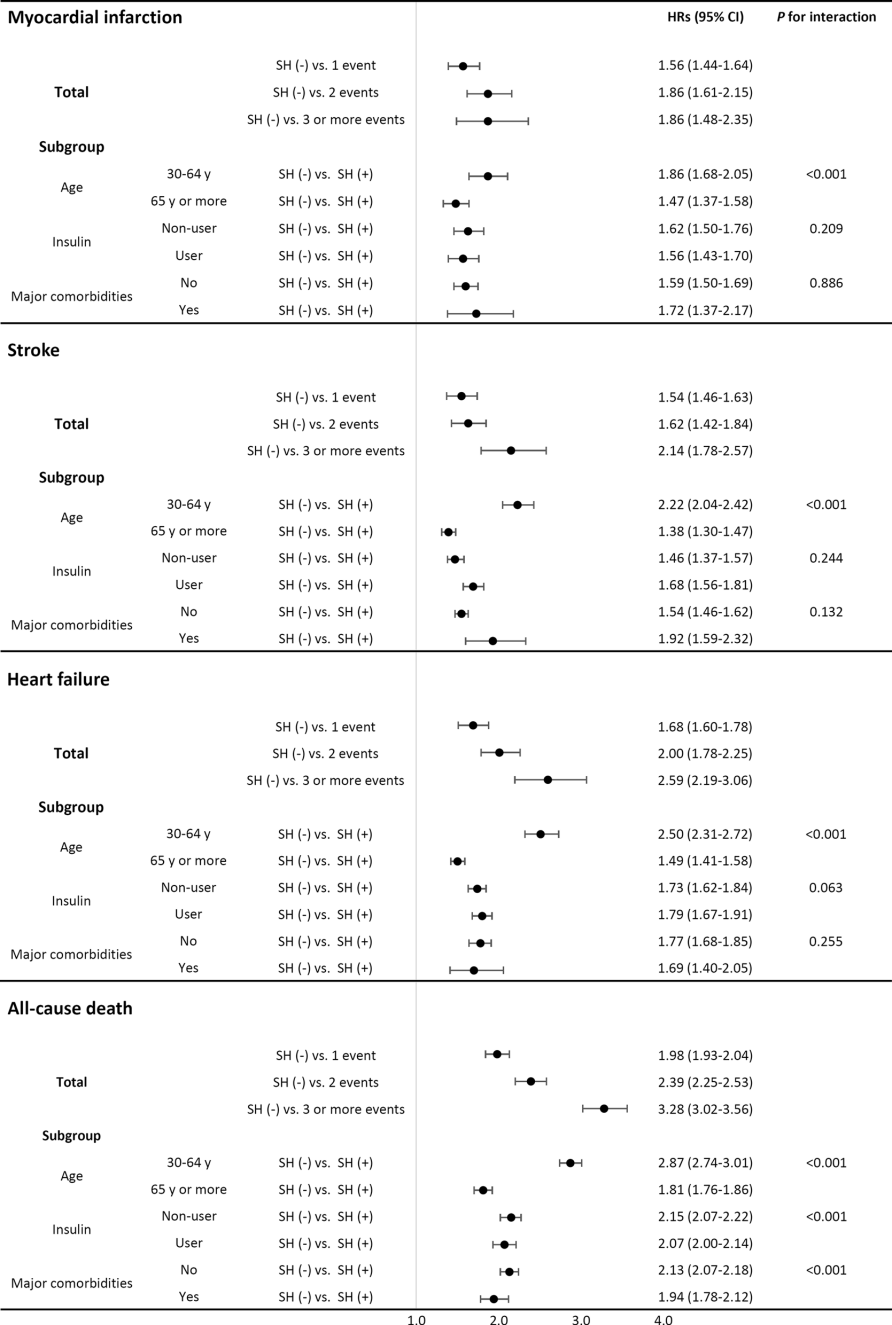
Forest plots for the association between SH and outcomes in total and subgroups. All HRs adjusted for covariates including age, sex, living place (urban or rural), income level, anti-diabetic drugs, the presence of hypertension, dyslipidemia, and major comorbidities (malignancy, liver cirrhosis, ESRD, and COPD)

During the follow-up period, 46,283 participants (3.0%) experienced a myocardial infarction event, and the incidence of myocardial infarction was 6.34 per 1000 patient-years. In addition, the number of stroke events was 60,176 (3.8%), and the number of heart failure events was 54,330 (3.5%). The incidence of stroke and heart failure was 7.21 and 6.60 per 1000 patient-years, respectively. A total of 153,036 participants (9.8%) died during about the 6-year follow-up period, representing a mortality incidence ratio of 17.27 per 1000 patient-years. The incidence of all outcomes tended to be higher in the group that experienced SH episodes at baseline (Table 2, Fig. 3).

**Table 2:** Crude and adjusted HRs of cardiovascular outcomes and all-cause mortality

Outcome	Number of severe hypoglycemia	N	Event	Incidence rate (per 1000 patient-years)	HR (95% CI)			*P* for trend
					Crude	Sex- and age adjusted	Multivariable^a^	
Myocardial infarction	0	1,548,437	58,529	6.7	Reference	Reference	Reference	< 0.001
	1	15,992	945	12.1	2.36 (2.21–2.51)	1.83 (1.72–1.96)	1.56 (1.46–1.64)	
	2	2543	182	16.1	3.12 (2.70–3.61)	2.31 (1.99–2.67)	1.86 (1.61–2.15)	
	3 or more	1125	72	15.5	2.99 (2.97–3.77)	2.39 (1.90–3.01)	1.86 (1.48–2.35)	
Stroke	0	1,548,437	58,529	6.7	Reference	Reference	Reference	< 0.001
	1	15,992	1304	16.9	2.53 (2.40–2.67)	1.78 (1.68–1.88)	1.54 (1.46–1.63)	
	2	2543	228	20.2	3.03 (2.66–3.45)	1.97 (1.73–2.24)	1.62 (1.42–1.84)	
	3 or more	1125	115	24.9	3.74 (3.11–4.49)	2.69 (2.25–3.24)	2.14 (1.78–2.57)	
Heart failure	0	1,548,437	52,485	6.0	Reference	Reference	Reference	< 0.001
	1	15,992	1423	18.4	3.13 (2.97–3.30)	2.09 (1.99–2.21)	1.68 (1.60–1.78)	
	2	2543	283	25.2	4.33 (3.85–4.87)	2.66 (2.36–2.99)	2.00 (1.78–2.25)	
	3 or more	1125	139	30.5	5.31 (4.49–6.27)	3.65 (3.09–4.31)	2.59 (2.19–3.06)	
All-cause death	0	1,548,437	146,332	16.5	Reference	Reference	Reference	< 0.001
	1	15,992	5058	63.3	3.88 (3.77–3.99)	2.46 (2.39–2.53)	1.98 (1.93–2.04)	
	2	2543	1074	91.6	5.63 (5.31–3.98)	3.24 (3.05–3.44)	2.39 (2.25–2.53)	
	3 or more	1125	572	119.0	7.37 (6.79–8.00)	4.73 (4.35–5.13)	3.28 (3.02–3.56)	

^a^Adjusted for age, sex, living place (urban or rural), income level, anti-diabetic drugs, the presence of hypertension, dyslipidemia, and major comorbidities (malignancy, liver cirrhosis, ESRD, and COPD)

**Fig. 4:**
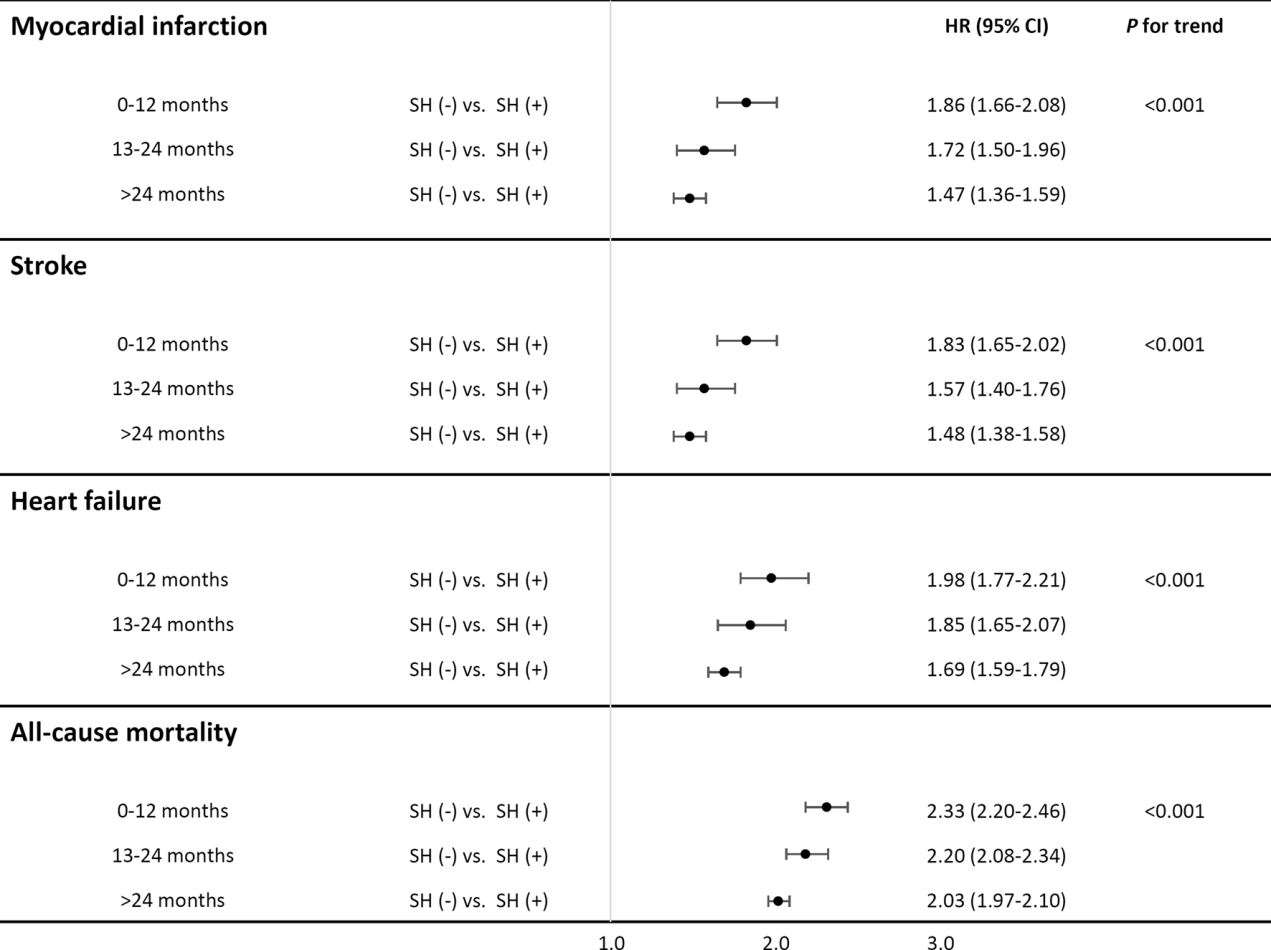
The association between SH and outcomes by follow-up time (0–12 months, 13–24 months, and > 24 months)

Compared with the group without SH, those with three or more SH episodes demonstrated a HR of 2.99 for myocardial infarction, 3.74 for stroke, 5.31 for heart failure, and 7.37 for all-cause mortality in the crude model. Further adjustment for multiple factors attenuated the associations; however, it did not change the significance of these relationships. Experiencing three or more SH episodes was associated with an approximately twofold higher risk of each type of cardiovascular event and a 3.28-fold higher risk of all-cause mortality. A dose–response relationship between the number of SH and all main outcomes was observed (*P* for trend < 0.001, Table 2, Fig. 3). In most of the outcomes, the hazard ratios for three or more SH episodes were higher than for one or two SH events. We examined the findings by subgroups of age, the use of insulin, and major comorbidities. In the subgroup analyses, there were no changes in the main relationship between the number of SH episodes and CVD or all-cause mortality (Fig. 3). All risks for cardiovascular disease and mortality were highest within 1 year of the index date and showed decreasing trends with follow-up (Fig. 4).

A sensitivity analysis was performed that included only the 797,544 participants who underwent a national health examination. The baseline characteristics for the sensitivity analysis are shown in Additional file 1: Table S1. Results for the Cox proportional hazard regression model are similar in all models after adding and adjusting further variables that could only be obtained from the medical health check-up database (Additional file 1: Table S2). Another sensitivity analysis excluding the subjects who experienced SH at least 2 years before the time of occurrence of main outcomes was performed to determine the effect of the SH event during the follow-up period on the relationship between SH and outcomes. Analyses after exclusion demonstrate that SH events which occurred during the follow-up period did not have a significant effect on the main relationship (Additional file 1: Table S3). A competing risk analysis that included mortality as a competing risk also resulted in a similar outcome as the main result (Additional file 1: Table S4).

## Discussion

Our study demonstrated the relationship between prior SH and increased risk of CVD and CVD-related mortality, which exhibited a dose–response relationship, wherein subjects who experienced more SH events had a higher risk of mortality and CVD than those who experienced one SH event or those without an SH event. In addition, our study demonstrated the temporal relationship between SH and CVD outcomes or all-cause mortality. These results remained consistent in the subgroup whether subjects have high or low CVD risk factors.

### Cardio- and cerebrovascular effect of SH

Hypoglycemia can be fatal; many previous studies have demonstrated that SH was associated with increased mortality in patients with type 1 and type 2 diabetes [9, 22–25]. One major hypothesis explaining the linkage between SH and increased mortality risk is that SH increases CVD risk [17, 26]. This hypothesis is plausible, because SH has been known to progress the atherogenic state by promoting catecholamine hypersecretion, proinflammatory cytokines, and platelet aggregation [26–28]. Additionally, the long-term cardiovascular effect on hypoglycemia can lead to increased endothelial dysfunction and a proinflammatory state, further contributing to atherosclerosis [29]. Hypoglycemia increases myocardial susceptibility to post-ischemic reperfusion injury and reduces the patient’s ability for ischemic preconditioning [30]. There may be another potential link between hypoglycemia and heart failure through subclinical myocardial damage because the myocardium may be directly damaged by the low glucose levels [31].

The relationship between SH and CVD outcomes or mortality are supported by many large randomized clinical trials, cohort studies, and meta-analyses [10, 12, 18, 23, 32–36]. However, some post hoc analyses of large clinical trials have raised doubts regarding whether there is a direct pathophysiological link between hypoglycemia and CVD outcomes. In the Action in Diabetes and Vascular Disease: Preterax and Diamicron MR Controlled Evaluation (ADVANCE) trial, the risk of non-cardiovascular disease (i.e., gastrointestinal or respiratory diseases), which seemed to have no relation to SH, also significantly increased in the SH group [23]. The result of the Trial Evaluating Cardiovascular Outcomes with Sitagliptin (TECOS) trial did not show a significant result when fully adjusted [33]. The double-blind Trial Comparing Cardiovascular Safety of Insulin Degludec vs Insulin Glargine in Patients with Type 2 Diabetes at High Risk of Cardiovascular Events (DEVOTE) 3 trials showed borderline significance of the relationship between SH and major cardiovascular outcomes [37]. Based on the previous inconsistent results, it has been argued that SH is merely a risk marker for CVD rather than a direct cause of CVD. SH usually occurs in advanced type 2 diabetes, and patients who experienced SH tend to have a higher cardiovascular risk. Therefore, it is considered that the association of SH with CVD would be related to reverse causation by the confounding factors rather than direct causality. It is difficult to clearly distinguish whether there is direct causality between SH and CVD. To prove causality clearly, an intervention study that was designed to compare the group with deliberately induced SH and without SH is needed [38]. However, this is ethically impossible. Instead, we can check several factors to assess causality, such as the strength, temporality, dose–response, consistency, and biological plausibility of the relationship [39]. Among them, this study demonstrated the dose–response, temporal relationship, and consistency of relationship between SH and CVD outcomes or mortality for determining causality.

Our analysis independently suggested the risk of SH for cerebrovascular outcome. The Atherosclerosis Risk in Communities (ARIC) cohort study showed a higher risk of CVD along with an increasing number of SH events for the outcomes of coronary heart disease, cardiovascular mortality, and all-cause mortality, not in the outcome of stroke. They explained the negative results of stroke and heart failure by the possibility of a small number of SH events or competing risks for mortality. Hypoglycemia also has a significant effect on the cerebral vascular system affecting systemic cardiovascular system by promoting inflammation, hypocoagulability, endothelial dysfunction, and oxidative stress [40]. Changing blood flow during hypoglycemia enhances the supply of glucose to the most vulnerable part of the brain which becomes permanent and persist during normoglycemia [41]. This permanent change can increase the risk of cerebral ischemia that leads to an increased risk of a cerebrovascular event [42]. In our study, the risk of stroke was significantly higher in the group with SH than the group without SH. The risk of stroke increased with the number of SH events and shorter follow-up periods after study initiation.

### Dose–response, temporal relationship and consistency of relationship between SH and CVD

There is controversy whether hypoglycemia-associated autonomic failure (HAAF) induced by recurrent episodes of hypoglycemia is an adaptive pathway or a maladaptive pathway for CVD [12]. Previous studies, the Action to Control Cardiovascular Risk in Type 2 Diabetes (ACCORD) and ADVANCE trials, showed a greater risk of CV death in the group with standard treatment with a lesser SH event than in the group with intensive treatment [18, 23]. VADT trials showed that despite more frequent serious results reported in the intensive treatment group, progression of coronary artery calcium scores after SH only developed in the standard treatment group [35]. It has been explained that repeated hypoglycemic episodes can induce HAAF, attenuate neuroendocrine secretion or the autonomic system, and lead to a weakened response to subsequent SH episodes [43]. However, in our real-world analysis, there was a clear dose–response relationship between the number of SH episodes and all CVD outcomes. This significant relationship remained after additional adjustment for standardized health check-up data, such as fasting plasma glucose, lipid profiles, and blood pressure, which can strongly affect CVD development and mortality.

Although it is generally accepted that hypoglycemia acutely affects CVD, there is limited evidence on the long-term effect of SH on the development of CVD. Results from the ADVANCE and the Examination of Cardiovascular Outcomes with Alogliptin versus Standard of Care (EXAMINE) trials showed no significant temporal response in the relationship between SH and CVD [23]. In contrast to these trials, the Liraglutide Effect and Action in Diabetes: Evaluation of Cardiovascular Outcome Results (LEADER) trial and case–control study from Taiwan demonstrated that there was a temporality between SH and CVD, and the risk of CVD outcomes and mortality was the highest shortly after SH events, and the risk remained up to 5 years, which is consistent with our results [36, 44]. Our study demonstrated that the risk of CVD outcomes is the highest within 1 year of the index date, and the risk of outcomes tends to decrease over time. The association between SH and CVD outcomes remained significant in the subgroup analyses, especially in the group with a low risk for CVD. The relationship between SH and CVD was augmented in the group with young subjects. In contrast with older patients with diabetes, younger patients have lower numbers of major cardiovascular risk factors. Severe hypoglycemia can have a much greater impact on the development of cardiovascular disease in young patients.

### Practical implications of causality between SH and CVD

The causality between SH and CVD has practical implications for selecting anti-diabetic agents. If there is a direct causality in the relationship between SH and CVD, anti-diabetic agents with a low risk for SH should be recommended preferentially to reduce the risk of CVD in patients with type 2 diabetes. However, there is still yet insufficient evidence to support this recommendation. A network meta-analysis of clinical trials suggested that the ranking of anti-diabetic agents for major adverse cardiovascular events was well correlated linearly with those for SH [45]. Another meta-analysis suggested that the beneficial effect for CVD of a glucagon-like peptide-1 (GLP-1) receptor agonist can be linked to a lowering effect of the risk for hypoglycemia [46]. On the other hand, the Empagliflozin Cardiovascular Outcome Event Trial in Type 2 Diabetes Mellitus Patients–Removing Excess Glucose (EMPA–REG), the Canagliflozin Cardiovascular Assessment Study (CANVAS), and the Trial to Evaluate Cardiovascular and Other Long-term Outcomes with Semaglutide in Subjects with Type 2 Diabetes (SUSTAIN-6) trials, which reported about the surprising CV benefit of sodium-glucose transport protein 2 (SGLT2) inhibitor and GLP-1 receptor agonist, demonstrated that there were no differences in the number of SH events between the group with treatment and the group with placebo [47–49], and that there was no treatment interaction between SH and CVD [44]. Further studies on the risk comparison among anti-diabetic agents including head-to-head clinical trial will be required.

### Strengths and limitations

There are some considerations in interpreting clinical and observational studies about SH. In the previous clinical trials, the interpretation of the association between hypoglycemia and CVD was limited, as they were not primarily designed to analyze the effect of hypoglycemia. In addition, the subjects of these clinical trials were recruited from high-risk CVD populations and were thus less representative of the general population. Moreover, the trials had a non-blind, randomized design, and subjects self-reported their hypoglycemic episodes; accordingly, the reporting may have differed in each group. Thus, it is feasible that there was more underreporting of hypoglycemia in the standard treatment group than in the intensive treatment group during the long-term trial. Lastly, the small number of SH events in each clinical trial is also a limitation in terms of the effect of SH. On the other hand, the epidemiologic investigations of SH also have several weaknesses, including relying on claim data for identifying SH events, missing some important variables related to SH or outcomes. Our study has also several limitations, similar to other epidemiologic studies. First, this study was based on a medical claims database. Therefore, the definition of hypoglycemia was not based on an independent measure of glucose; thus, misclassification may be possible. Second, our study relied on the medical claims and National Health database, which lack data on important variables for CVD in diabetes, such as the duration of disease; level of HbA1c; glycemic variability; dosage, type, and exposure duration of antidiabetic medications; cardiac autonomic neuropathy; or baseline electrocardiogram status, which may explain the increased risk of CVD outcomes and mortality in patients with SH events. Therefore, additional community- or hospital-based cohort studies that have standardized, high-quality data on clinical characteristics are needed. Nevertheless, the present study was conducted in a large nationwide population, and we were able to analyze a sufficient number of SH events and include patients with low CVD risk factors. The strength of our study is that all relevant medical data for the Korean population were included in the analyses. Furthermore, to the best of our knowledge, this is the largest population-based analysis to date regarding the association between recurrent SH and CVD.

## Conclusion

This study highlights the prognostic importance of prior history of SH on cardiovascular events and mortality. SH was strongly and positively associated with and exhibited a dose-dependent and temporal relationship with subsequent macrovascular morbidity and all-cause mortality. In patients who experienced SH, preventing the risk of CVD and mortality should be carefully considered in patients who are at a greater risk of hypoglycemia. In addition, the study illustrates the need for careful management and frequent monitoring of all individuals with type 2 diabetes to minimize the risk of hypoglycemia. However, more studies are needed to clarify whether prior SH is itself a real risk factor for CVD and mortality or whether it is a marker for another factor, such as cardiac autonomic neuropathy, which may be associated with increased mortality.

## Additional file

**Additional file 1: Table S1.** Baseline characteristics according to the number of severe hypoglycemia events of sensitivity analysis from the sub-cohort which included only the participants who had regular standardized medical checkups. **Table S2.** Crude and adjusted HRs of cardiovascular outcomes and all-cause mortality from the sub-cohort which included only the participants who had regular standardized medical checkup. **Table S3.** Crude and adjusted HRs of cardiovascular outcomes and all-cause mortality from the sub-cohort with exclusion of patients who experienced SH at least two years before the occurrence of main outcomes. **Table S4.** Competing risk analysis including mortality as a competing risk.

## Data Availability

The datasets generated and/or analyzed during the current study are available in the National Health Institute Database. (The researchers who are authorized to access the information can obtain anonymized health information from the database, https://nhiss.nhis.or.kr).

## Abbreviations

ACCORD: Action to Control Cardiovascular Risk in Diabetes
ADVANCE: Action in Diabetes and Vascular Disease: Preterax and Diamicron MR Controlled Evaluation
ARIC: the Atherosclerosis Risk in Communities
BMI: body mass index
CANVAS: Canagliflozin Cardiovascular Assessment Study
COPD: chronic obstructive pulmonary disease
CVD: cardiovascular disease
DEVOTE: double-blind Trial Comparing Cardiovascular Safety of Insulin Degludec vs Insulin Glargine in Patients with Type 2 Diabetes at High Risk of Cardiovascular Events
eGFR: estimated glomerular filtration rate
EMPA–REG: Empagliflozin Cardiovascular Outcome Event Trial in Type 2 Diabetes Mellitus Patients–Removing Excess Glucose
ESRD: end-stage renal disease
EXAMINE: Examination of Cardiovascular Outcomes with Alogliptin versus Standard of Care
GLP-1: glucagon like peptide-1
HAAF: hypoglycemia-associated autonomic failure
ICD-10: International Classification of Disease, 10th revision
LEADER: the Liraglutide Effect and Action in Diabetes: Evaluation of Cardiovascular Outcome Results
MDRD: Modification of Diet in Renal Disease Study Group equation
NHIS: National Health Insurance Service
ORIGIN: Outcome Reduction with Initial Glargine Intervention
SGLT2: sodium–glucose transport protein 2
SH: severe hypoglycemia
SUSTAIN: Trial to Evaluate Cardiovascular and Other Long-term Outcomes with Semaglutide in Subjects with Type 2 Diabetes
TECOS: Trial Evaluating Cardiovascular Outcomes with Sitagliptin
VADT: the Veterans Affairs Diabetes Trial
