# Supplementary material for: Severe hypoglycemia and the risk of cardiovascular disease and mortality in type 2 diabetes: a nationwide population-based cohort study
Source: Cardiovasc Diabetol. 2019 Aug 14;18:103. doi: 10.1186/s12933-019-0909-y (PMC6694505; doi:10.1186/s12933-019-0909-y)
Supplement: Supplementary file 1 — Additional file 1: Table S1. Baseline characteristics according to the number of severe hypoglycemia events of sensitivity analysis from the sub-cohort which included only the participants who had regular standardized medical checkups. Table S2. Crude and adjusted HRs of cardiovascular outcomes and all-cause mortality from the sub-cohort which included only the participants who had regular standardized medical checkup. Table S3. Crude and adjusted HRs of cardiovascular outcomes and all-cause mortality from the sub-cohort with exclusion of patients who experienced SH at least two years before the occurrence of main outcomes. Table S4. Competing risk analysis including mortality as a competing risk. [file 12933_2019_909_MOESM1_ESM.docx]

Table S1. Baseline characteristics according to the number of severe hypoglycemia events of sensitivity analysis from the sub-cohort which included only the participants who had regular standardized medical checkups.

|  | Number of severe hypoglycemia events | | |  |
| --- | --- | --- | --- | --- |
|  | 0 | 1 | 2 or more | *P* value |
| Total | 791,131 | 5,514 | 899 |  |
| AGE | 59.9 ± 10.3 | 65.8 ± 10.4 | 67.0 ± 10.9 | <0.001 |
| Age (%) |  |  |  | <0.001 |
| 30–49 years | 160,337 (20.3) | 529 (9.6) | 78 (8.7) |  |
| 50–64 years | 385,245 (48.7) | 1,900 (34.5) | 275 (30.6) |  |
| 65– years | 245,549 (31.0) | 3,085 (56.0) | 546 (60.7) |  |
| Sex (male) | 443,321 (56.0) | 2,696 (48.9) | 470 (52.3) | <0.001 |
| Urban (%) | 358,109 (45.3) | 2,022 (36.7) | 293 (32.6) | <0.001 |
| BMI (kg/m^2^) | 25.0 ± 3.2 | 23.8 ± 3.4 | 22.9 ± 3.6 | <0.001 |
| Waist circumference (cm) | 85.6 ± 8.3 | 83.7 ± 8.7 | 82.1 ± 9.1 | <0.001 |
| Medication (%) |  |  |  |  |
| Insulin | 88,318 (11.2) | 2,046 (37.1) | 443 (49.3) | <0.001 |
| Sulfonylurea | 619,860 (78.4) | 4,307 (78.1) | 647 (72.0) | <0.001 |
| Metformin | 570,169 (72.1) | 3,934 (71.4) | 608 (67.6) | 0.006 |
| Meglitinide | 34,073 (4.3) | 473 (8.6) | 105 (11.7) | <0.001 |
| Thiazolidinedione | 107,809 (13.6) | 816 (14.8) | 119 (13.2) | 0.039 |
| DPP4 inhibitor | 72,055 (9.1) | 471 (8.5) | 73 (8.1) | 0.205 |
| Acarbose | 168,961 (21.4) | 1,881 (34.1) | 363 (40.4) | <0.001 |
| Hypertension (%) | 430,482 (54.4) | 3,631 (65.9) | 612 (68.1) | <0.001 |
| Dyslipidemia (%) | 310,366 (39.2) | 1,968 (35.7) | 292 (32.5) | <0.001 |
| COPD (%) | 25525 (3.2) | 210 (3.8) | 33 (3.7) | <0.001 |
| ESRD (%) | 190 (0.0) | 13 (0.2) | 4 (0.4) | <0.001 |
| Malignancy (%) | 6907 (0.9) | 86 (1.6) | 13 (1.5) | <0.001 |
| Liver cirrhosis (%) | 1805 (0.2) | 17 (0.3) | 3 (0.3) | <0.001 |
| Socioeconomic status (%) |  |  |  | 0.774 |
| Lower 30% | 235,585 (29.8) | 1,669 (30.3) | 276 (30.7) |  |
| Mid 40% | 256,461 (32.4) | 1,766 (32.0) | 277 (30.8) |  |
| Upper 30% | 299,085 (37.8) | 2,079 (37.7) | 346 (38.5) |  |
| Estimated GFR (mL/min/1.73m^2^) | 83.6 ± 35.6 | 73.5 ± 44.2 | 66.4 ± 30.7 | <0.001 |
| Chronic kidney disease | 99,763 (12.6) | 1,761 (31.9) | 391 (43.5) | <0.001 |
| Smoking status (%) |  |  |  | <0.001 |
| Never smoker | 475,868 (60.2) | 3,738 (67.8) | 593 (66.0) |  |
| Former smoker | 146,266 (18.4) | 797 (14.4) | 129 (14.3) |  |
| Current smoker | 168,997 (21.4) | 979 (17.8) | 177 (19.7) |  |
| Alcohol consumption (%) |  |  |  | <0.001 |
| None | 498,821 (63.1) | 4,109 (74.5) | 675 (75.1) |  |
| Moderate consumption | 236,217 (29.9) | 1,099 (19.9) | 171 (19.0) |  |
| Heavy drinking | 56,093 (7.1) | 306 (5.6) | 53 (5.9) |  |
| Fasting glucose (mmol/L) | 7.87 ± 2.64 | 7.49 ± 3.02 | 7.61 ± 3.31 | <0.001 |
| Hypertension (%) | 492,203 (62.2) | 3,939 (71.4) | 666 (74.1) | <0.001 |
| SBP (mmHg) | 128.6 ± 15.5 | 129.0 ± 16.7 | 128.2 ± 17.6 | 0.164 |
| DBP (mmHg) | 78.2 ± 9.8 | 77.0 ± 10.2 | 76.3 ± 10.1 | <0.001 |
| Dyslipidemia (%) | 368,488 (46.6) | 2,323 (42.2) | 335 (37.4) | <0.001 |
| Total cholesterol (mmol/L) | 4.95 ± 1.04 | 4.75 ± 1.08 | 4.65 ± 1.07 | <0.001 |
| Triglyceride (mmol/L) | 1.62 (1.62-1.62) | 1.46 (1.44-1.48) | 1.35 (1.30-1.40) | <0.001 |
| HDL-cholesterol (mmol/L) | 1.32 ± 0.50 | 1.32 ± 0.52 | 1.33 ± 0.51 | 0.866 |
| LDL-cholesterol (mmol/L) | 2.80 ± 1.13 | 2.70 ± 1.30 | 2.62 ± 1.01 | <0.001 |

Values are presented as percentage or mean ± standard deviation.

COPD, chronic obstructive pulmonary disease; ESRD, end-stage renal disease; GFR, glomerular filtration rate; SBP, systolic blood pressure; DBP, diastolic blood pressure

Table S2. Crude and adjusted HRs of cardiovascular outcomes and all-cause mortality from the sub-cohort which included only the participants who had regular standardized medical checkup.

| Outcome | Number of  severe hypoglycemia | N | Event | Incidence rate  (per 1000 person-years) | HR (95%CI) | | |
| --- | --- | --- | --- | --- | --- | --- | --- |
|  |  |  |  |  | Crude | Sex- and age-adjusted | Multivariable* |
| Myocardial  infarction | 0 | 791,131 | 20,509 | 4.48 | Reference | Reference | Reference |
|  | 1 | 5,514 | 292 | 9.91 | 2.22 (1.98-2.49) | 1.78 (1.59-2.00) | 1.49 (1.35-1.64) |
|  | 2 or more | 899 | 67 | 15.03 | 3.37 (2.67-4.30) | 2.57 (2.03-3.27) | 1.85 (1.50-2.27) |
| Stroke | 0 | 791,131 | 25,889 | 5.67 | Reference | Reference | Reference |
|  | 1 | 5,514 | 426 | 14.59 | 2.59 (2.35-2.85) | 1.99 (1.80-2.19) | 1.42 (1.26-1.60) |
|  | 2 or more | 899 | 93 | 21.11 | 3.78 (3.08-4.63) | 2.71 (2.21-3.32) | 1.85 (1.45-2.37) |
| Heart failure | 0 | 791,131 | 20,695 | 4.51 | Reference | Reference | Reference |
|  | 1 | 5,514 | 389 | 13.23 | 2.99 (2.70-3.30) | 2.25 (2.03-2.49) | 1.53 (1.38-1.69) |
|  | 2 or more | 899 | 105 | 23.73 | 5.44 (4.49-6.59) | 3.83 (3.16-4.64) | 2.19 (1.80-2.67) |
| All-cause death | 0 | 791,131 | 46,398 | 10.02 | Reference | Reference | Reference |
|  | 1 | 5,514 | 1,063 | 35.28 | 3.58 (3.36-3.80) | 2.75 (2.59-2.92) | 1.79 (1.68-1.90) |
|  | 2 or more | 899 | 271 | 58.83 | 6.04 (5.36-6.81) | 4.33 (3.84-4.87) | 2.50 (2.21-2.83) |

*Adjusted for age, sex, living place (urban or rural), income level, anti-diabetic drugs, the presence of hypertension, dyslipidemia, and major comorbidities, smoking, alcohol, fasting plasma glucose, LDL-cholesterol, chronic kidney disease

SH, severe hypoglycemia; congestive heart failure, congestive heart failure

Table S3. Crude and adjusted HRs of cardiovascular outcomes and all-cause mortality from the sub-cohort with exclusion of patients who experienced SH at least two years before the occurrence of main outcomes.

| Outcome | Number of  severe hypoglycemia | N | Event | Incidence rate  (per 1000 person-years) | HR (95%CI) | | |
| --- | --- | --- | --- | --- | --- | --- | --- |
|  |  |  |  |  | Crude | Sex- and age adjusted | Multivariable* |
| Myocardial  infarction | 0 | 1,529,675 | 43,795 | 5.05 | 1 | 1 | 1 |
|  | 1 | 15,594 | 893 | 11.51 | 2.26 (2.13-2.44) | 1.77 (1.66-1.89) | 1.51 (1.41-1.61) |
|  | 2 or more | 3,581 | 243 | 15.22 | 3.02 (2.66-3.42) | 2.28 (2.01-2.58) | 1.82 (1.60-2.06) |
| Stroke | 0 | 1,529,675 | 56,674 | 6.55 | 1 | 1 | 1 |
|  | 1 | 15,594 | 1,242 | 16.16 | 2.48 (2.34-2.62) | 1.73 (1.64-1.83) | 1.50 (1.42-1.59) |
|  | 2 or more | 3,581 | 335 | 21.12 | 3.25 (2.92-3.61) | 2.16 (1.94-2.41) | 1.75 (1.57-1.95) |
| Heart failure | 0 | 1,529,675 | 50,268 | 5.78 | 1 | 1 | 1 |
|  | 1 | 15,594 | 1,372 | 17.79 | 3.14 (2.97-3.31) | 2.09 (1.98-2.21) | 1.68 (1.59-1.77) |
|  | 2 or more | 3,581 | 411 | 26.10 | 4.67 (4.24-5.14) | 2.95 (2.67-3.25) | 2.17 (1.97-2.39) |
| All-cause death | 0 | 1,529,675 | 137,426 | 15.64 | 1 | 1 | 1 |
|  | 1 | 15,594 | 4,710 | 59.31 | 3.83 (3.72-3.94) | 2.41 (2.34-2.49) | 1.95 (1.89-2.01) |
|  | 2 or more | 3,581 | 1,567 | 95.26 | 6.19 (5.89-6.50) | 3.65 (3.47-3.84) | 2.64 (2.51-2.77) |

*Adjusted for age, sex, living place (urban or rural), income level, anti-diabetic drugs, the presence of hypertension, dyslipidemia, and major comorbidities, smoking, alcohol, fasting plasma glucose, LDL-cholesterol, chronic kidney disease

Table S4. Competing risk analysis including mortality as a competing risk

| Outcome | Number of severe hypoglycemia | HR (95%CI) | | |
| --- | --- | --- | --- | --- |
|  |  | Crude | Sex- and age adjusted | Multivariable^*^ |
| Myocardial  infarction | 0 | Reference | Reference | Reference |
|  | 1 | 2.06 (1.93-2.19) | 1.61 (1.51-1.72) | 1.39 (1.30-1.49) |
|  | 2 or more | 2.42 (2.14-2.74) | 1.85 (1.63-2.09) | 1.51 (1.33-1.71) |
| Stroke | 0 | Reference | Reference | Reference |
|  | 1 | 2.21 (2.09-2.33) | 1.55 (1.47-1.64) | 1.36 (1.29-1.44) |
|  | 2 or more | 2.55 (2.29-2.83) | 1.71 (1.54-1.91) | 1.44 (1.28-1.59) |
| Heart failure | 0 | Reference | Reference | Reference |
|  | 1 | 2.69 (2.55-2.84) | 1.81 (1.71-1.91) | 1.47 (1.40-1.56) |
|  | 2 or more | 3.53 (3.20-3.89) | 2.25 (2.04-2.49) | 1.69 (1.53-1.87) |

*Adjusted for age, sex, living place (urban or rural), income level, anti-diabetic drugs, the presence of hypertension, dyslipidemia, and major comorbidities, smoking, alcohol, fasting plasma glucose, LDL-cholesterol, chronic kidney disease
